# Understanding the Degradation of Hominid Gene Control

**DOI:** 10.1371/journal.pcbi.0020019

**Published:** 2006-03-31

**Authors:** Peter D Keightley, Martin J Lercher, Adam Eyre-Walker

Recently, two groups have examined the level of sequence constraint in noncoding DNA flanking mammalian genes, and appear to have found conflicting results. By comparing 500-bp blocks in mice and rats, we found that mean nucleotide divergence within 2 kb of the start and stop codons of protein-coding genes is substantially lower than that of introns, and decreases when approaching the coding sequence [1]. If nucleotide changes within introns are largely free from selection, this implies that noncoding blocks close to genes evolved under selective constraints, presumably because they contain gene expression control regions. In contrast, we find that upstream sequences in hominids do not evolve slower than introns, while downstream regions are under about half of the constraint seen in murids [1].

By analysing a similar set of noncoding DNA sequences, Bush and Lahn also found that the mean level of selective constraints in upstream regions between humans and chimpanzees is very low. However, their slightly more complex main analysis was to search for 16-bp sequences within upstream regions that are strongly conserved between humans, mice, and either dogs or chickens. They then examined the divergence between humans and chimpanzees at the flanking nucleotides, finding substantially reduced divergence compared with the genomic mean. This demonstrated selective constraints at certain upstream sequences in hominids. An analogous analysis of mouse–rat sequences showed that the selective constraints are about twice as strong in murids as in hominids [2].

These two findings—on one hand, a near absence of selective constraints in blocks upstream of hominid genes [1], and on the other, evidence for strong selective constraints in these regions [2]—appear to contradict each other. How can we square the two sets of results? The answer is rather simple—windows with high conservation scores are relatively rare, and they contribute little to the mean calculated over 500-bp windows (unfortunately, Bush and Lahn do not tell us the fraction of 5′ alignments within high conservation scores).

Bush and Lahn also suggest that the apparent discrepancy “likely results from the fact that in large 500-bp blocks, functional elements that are under constraint are mixed with large sections of nonfunctional DNA, which are not under constraint” [2]. We believe that this interpretation, while formally correct, obscures important and interesting information that can be gained from combining the two studies. Some sequences outside the conserved 16-mers identified by Bush and Lahn are also likely to be functional, since the same 500-bp regions (largely “nonfunctional” according to Bush and Lahn) show strong evidence of evolutionary constraints between mice and rats [1].

Bush and Lahn also note that constraint, on either side of conserved windows, is greater in murids than in hominids. However, they observe a much smaller difference than that seen in our analysis. This is deceptive because by concentrating attention on regions that are conserved between humans, mice, and dogs, they ignore the fact that there might be many more highly conserved regions in murids than there are in hominids.

In summary, there is no conflict between our results and those of Bush and Lahn; they concentrate their attention on a preselected subset of the sites we considered and so have a different perspective on the problem. What is clear from both studies is that there is a qualitative difference in the level of conservation in the 5′ flanking sequences between murids and hominids. We have argued that this is likely to be due to the fixation of slightly deleterious mutations in hominids that are otherwise selectively eliminated in rodents. Differences in constraints between hominids and murids demonstrate that the overwhelming majority of changes at upstream regulatory sites have only small effects on fitness. This has counterintuitive consequences: to obtain a comprehensive list of human regulatory sites, it might be better to examine conservation in murid rather than hominid genomes.
